# Driving and mobile phone use: Work addiction predicts hazardous but not excessive mobile phone use in a longitudinal study of young adults

**DOI:** 10.1556/2006.2024.00007

**Published:** 2024-03-08

**Authors:** Bernadette Kun, Borbála Paksi, Andrea Eisinger, Gyöngyi Kökönyei, Zsolt Demetrovics

**Affiliations:** 1 Institute of Psychology, ELTE Eötvös Loránd University, Budapest, Hungary; 2 Institute of Education, ELTE Eötvös Loránd University, Budapest, Hungary; 3 NAP3.0 – SE Neuropsychopharmacology Research Group, Hungarian Brain Research Program, Semmelweis University, Budapest, Hungary; 4 Department of Pharmacodynamics, Faculty of Pharmacy, Semmelweis University, Budapest, Hungary; 5 Centre of Excellence in Responsible Gaming, University of Gibraltar, Gibraltar, Gibraltar

**Keywords:** work addiction, problematic mobile phone use, driving, anxiety, worry, rumination

## Abstract

**Background and objectives:**

Work addiction (WA), characterized by dimensions such as overcommitment, difficulties in detachment from work, and work-life imbalance, is presumed to be associated with increased smartphone usage, even during risky activities like driving. The study investigated the connection between WA and future problematic and hazardous smartphone use, considering personality factors: anxiety, rumination, and worry.

**Methods:**

A three-wave longitudinal study (*N* = 1,866) was conducted from March to July 2019, June to September 2020, and June to November 2021, involving a representative sample of 18-34-year-old residents in Hungary's capital. The study employed Hungarian versions of the Bergen Work Addiction Scale, Problematic Mobile Phone Use Questionnaire, Ruminative Response Scale, Anxiety subscale of the Brief Symptom Inventory 18, and Penn-State Worry Questionnaire. Additionally, author-developed questions on mobile phone use while driving were included.

**Results:**

At baseline, those at risk for WA showed more frequent mobile phone use while driving at both time points 2 and 3 compared to the non-risk group. Path analyses revealed rumination, anxiety at time 1, and worry at time 2 as significant mediators between baseline WA and mobile phone use while driving at time 3. However, when analyzing all three mediators together, only anxiety at time 1 and worry at time 2 remained significant.

**Discussion and conclusion:**

This study demonstrates that WA predicts future mobile phone use while driving through mediation by anxiety and worry. Our findings add to the growing evidence highlighting the detrimental aspects of WA, emphasizing the need for improved prevention and treatment strategies.

## Introduction

Work addiction has garnered increasing attention within the realm of behavioral addictions, experiencing a notable upsurge in research. Although it is not currently recognized within diagnostic manuals as an official mental disorder, the high prevalence rates (Andreassen et al., 2014; Berk, Ebner, & Rohrbach-Schmidt, 2022; Kang, 2020) and the numerous adverse factors linked with this issue (Clark et al., 2016) emphasize the significance of delving deeper into its psychological underpinnings and resulting ramifications.

The concept of work addiction has been approached through various models, such as those proposed by Schaufeli et al. (2008), distinguishing between excessive and compulsive work, and Griffiths (2005) with the component model of addictions outlining seven primary symptoms. Clark et al.'s (2020) multidimensional model integrates emotional, cognitive, motivational, and behavioral factors. These models, along with earlier theories (Ng et al., 2007; Oates, 1971; Robinson, 2007), emphasize the escalating negative consequences of excessive work, impacting physical health, health behaviors, psychological well-being, social relationships, and career (Burke & MacDermid, 1999; Clark et al., 2020; Kun et al., 2023; Matsudaira et al., 2013; Salanova et al., 2016). Moreover, evidence suggests a correlation between work addiction and increased use of substances like alcohol, stimulants, and tranquilizers (Kun et al., 2023; Péter et al., 2023; Salanova et al., 2016), linking it with mental health concerns and other behavioral addictions.

Limited knowledge exists about potential comorbidity with other behavioral addictions, but data is available concerning excessive internet usage and smartphone usage (Cheung et al., 2022; Quinones et al., 2016). Workaholics not only employ their smartphones more extensively than non-workaholics but also exhibit a higher frequency of usage during the evening hours after work, consequently leading to heightened sleep-related issues. This study specifically examined smartphone usage, yet it also anticipated a correlation between work addiction and overall mobile phone use. While both devices share the basic functions of making calls and sending messages, smartphones offer additional features such as internet connectivity, app downloading capabilities, and an enhanced camera.

If we posit that harmful outcomes play a pivotal role in work addiction, it follows that the detrimental use of mobile phones and smartphones is also likely prevalent. This assumption is easily conceivable, given that individuals prone to work addiction often overextend themselves, struggle to disengage from their work, and encounter difficulty in separating professional responsibilities from their personal lives (Aziz, Adkins, Walker, & Wuensch, 2010; Robinson, 2007). Situations where excessive mobile phone or smartphone usage leads to problems can also emerge. Additionally, individuals with work addiction are characterized by negative affectivity, anxiety, and rumination (Clark et al., 2016; Kun, Takacs, et al., 2020a; Kun, Urbán, et al., 2020b), which could serve not only as precursors to work addiction but also as its consequences. For instance, heightened stress, chronic fatigue, and insomnia can exacerbate negative psychological symptoms (Virtanen et al., 2007; Williamson et al., 2005). Hence, it remains plausible that work addiction exhibits a stronger connection with excessive and problematic smartphone and mobile phone usage due to heightened anxiety, worry, and rumination (i.e., perseverative cognitions) linked to work-related concerns. This could lead to situations where individuals not only cannot distance themselves from their devices but also employ them in risky scenarios. This might encompass using the mobile phone or smartphone while driving, including actions like not using a hands-free device, texting, or browsing social networking platforms.

In this study, we aim to explore this relationship within a longitudinal framework. Our objective is to ascertain whether work addiction correlates with subsequent patterns of excessive and problematic mobile phone usage, even while driving. Furthermore, we hypothesize that the link between work addiction and subsequent problematic mobile phone use is mediated by anxiety and perseverative cognitions, namely rumination and worry. We hypothesized that work addiction at time T1 would predict both excessive mobile phone use and mobile phone use while driving at time T3. Furthermore, we anticipated that higher levels of rumination and anxiety at time T1, along with worry at time T2, would mediate this relationship. To our knowledge, such research, particularly employing a longitudinal design, remains scarce. Therefore, this study addresses an existing knowledge gap by exploring whether work addiction 1) predicts the emergence of other behavioral addictions (i.e., problematic mobile phone use) later in life, and 2) correlates with enduring maladaptive behaviors, specifically excessive and problematic mobile phone use. Moreover, 3) the examination of mediating factors aims to determine whether perseverative cognitions contribute to the association between work addiction and problematic mobile phone use. These connections have yet to be explored, but their investigation could significantly contribute to our understanding of work addiction as a phenomenon.

## Methods

### Participants and procedure

This study leveraged data collected in waves 1–3 of a longitudinal study to investigate the enduring patterns of addictive behaviors and their associated psychological factors. Specifically, the focus was on the connection between work addiction and mobile phone use, with other addictive disorders excluded from the analysis. The study targeted young adults residing in Budapest, Hungary, and the sample comprised individuals born between 1984 and 2000 who had a valid Budapest address according to the Ministry of Interior's records. The sample selection employed a one-stage random sampling method, stratified by age groups (18–24 and 25–34) and place of residence (districts). Data for waves 1–3 were collected during specific timeframes: March to July 2019, June to September 2020, and June to November 2021. Data collection was conducted through a combination of in-person interviews and self-report assessments. In waves 2 and 3, participants had the option to participate online through computer-assisted web interviews. During the second wave, 57 respondents (2%) opted for the online option, while in the third wave, 7 respondents (0.2%) chose to participate online. All individuals in the ultimate sample provided informed consent for voluntary participation in the study, which received ethical approval from the Scientific and Research Ethics Committee of the Medical Research Council.

To ensure consistent communication with the participants, we collect and process the following information: (a) the subject's full name and contact details (residential address, online contact information); (b) the full name and contact details of an alternative person chosen by the subject, to be used if the subject cannot be reached for any reason. The personal data gathered during this research will be strictly utilized for contact purposes only. It will not be shared with any third parties and will only be accessible to the research participants or their designated contacts to whom the data pertains. The questionnaire ensures anonymity as it does not contain any information enabling the identification of individual respondents. Nonetheless, in order to facilitate the linkage of data collected across various waves of data collection, each participant is required to create a six-character-long identifier. This identifier is formulated as follows: 1) The second letter of the participant's first name at birth; 2) The third letter of the participant's current first name; 3) The fourth character of the participant's date of birth; 4); The eighth character of the participant's date of birth; 5) The second character of the mother's maiden surname; and 6) The third character of the participant's mother's first maiden name. The identifier generated by the algorithm above possesses a specific trait: it ensures that from the data of a single person, the same identifier can consistently be generated (minimizing the likelihood of repetition within the intended sample size). This feature allows for the linkage of data collected across various collection phases while ensuring the identifier remains non-decomposable and devoid of any information that could lead to the identification of individuals participating in the research.

One of the key priorities within the sampling strategy was to ensure that the net sample size at each wave could afford a theoretical margin of error of up to 2.2% at a confidence level of 95% for analyzing data related to the target population. The initial sample size was determined by considering both the attrition rate and age-standardized sample attrition patterns observed in national longitudinal studies (Bartus, 2015), along with the achieved net/gross sample size ratio through the utilization of a surrogate sample in the OLAAP 2015 study concerning the young adult population in the capital city (Paksi et al., 2021).

A total of 3,914 individuals participated in one or more of the three waves (waves 1–3) of the study. Within this group, 3,890, 2,801, and 2,874 respondents completed the questionnaires for waves 1, 2, and 3, respectively. This translates to the following response rates for each wave: in the first wave, from a gross sample size of 4,500 people, the 3,890 participants represent a response rate of 86%. In the second wave, we retained 72% of the initial participants, and in the third wave, we achieved a response rate of 77%. To provide an overview, 2,563 participants engaged in all three waves of the survey, with females accounting for 50.81% (*N* = 1,302) of this group. Their average age in wave 1 stood at 27.05 years (*SD* = 4.77). To arrive at the final sample for analysis, three inclusion criteria were applied. First, participants had to be present in all three waves of the survey. Second, they needed to report a minimum of 40 h of gainful employment per average week over the past year in all three waves. The rationale behind this criterion stemmed from numerous studies indicating a significant positive correlation between workload volume and the symptoms of work addiction (Atroszko, Pallesen, Griffiths, & Andreassen, 2017; Clark et al., 2016). It is assumed that individuals not engaged in full-time employment, specifically those working fewer than 40 h per week, are generally less likely to exhibit work addiction tendencies. Three, only individuals who report using a mobile phone in all three waves were included in the analysis. The exclusion criteria were as follows: 1) not being a resident of Budapest; 2) not born between 1984 and 2000; 3) not having worked at least 40 h on average per week in the past year, and 4) and not using a mobile phone. In the initial data collection wave, 18.2% of the sample did not maintain an average workweek of 40 h. Subsequently, in the second and third waves, 15.2 and 11.1% of individuals, respectively, were excluded from the final sample due to this criterion. In the first wave, 0.8% did not meet the criterion for mobile phone use, while in the second wave, it was 1.8%, and in the third wave, it reached 1.5%.

All these criteria yielded a final sample of 1,866 individuals, with females representing 49.9% (*N* = 932) of this subset. In wave 1, the average age was 27.81 (*SD* = 4.36), and the average weekly working hours were 42.10 (*SD* = 4.19), which were comparable to wave 2 (*M* = 41.71, SD = 3.98) and wave 3 (*M* = 41.57, *SD* = 3.73).

Due to a lack of responses from many participants, it became essential to analyze the pattern of missing data. To assess whether imputation was required, we employed Little's MCAR (Missing Completely at Random) test. The test results were significant, (*χ*^*2*^ = 3,593.64; *df* = 3,125; *p* < 0.001) suggesting that the missing data did not occur randomly. Consequently, we utilized imputation, employing the Expectation Maximization algorithm in SPSS to fill in the gaps in the data.

### Measures

#### Work addiction

Work addiction was assessed by the 7-item Bergen Work Addiction Scale (BWAS) across all three waves of the study (Andreassen, Griffiths, Hetland, & Pallesen, 2012; Orosz et al., 2016). The BWAS evaluates the seven fundamental components of work addiction (Griffiths, 2005; Griffiths & Karanika-Murray, 2012). Participants provided their responses using a 5-point scale, ranging from 0 (‘Not characteristic at all’) to 4 (‘Very characteristic’). For our analyses, we considered the total BWAS score for each wave, with higher scores indicating greater work addiction severity. A risk of work addiction was identified if an individual scored 3 (‘More characteristic’) or 4 (‘Very characteristic’) on at least four items of the BWAS, in accordance with the criteria set forth by Andreassen et al. (2012). The scale showed good internal reliability across all three waves (see Supplementary Table S1). The scale has a validated Hungarian adaptation, and we have utilized this particular version (Orosz et al., 2016).

#### Excessive mobile phone use

To assess excessive mobile phone use at T1, T2, and T3, we employed the Dependency subscale of the short version of the Problematic Mobile Phone Use Questionnaire (PMPUQ-SV; Lopez-Fernandez et al., 2018). The scale comprises five items (e.g., “I feel lost without my mobile phone.”), each requiring participants to self-assess on a five-point Likert scale (ranging from 1 = ‘I strongly agree’ to 4 = ‘I strongly disagree’). Note that items 1 and 3 are reverse-scored. A higher score indicates a greater difficulty in functioning without a mobile phone. The scale showed good internal consistency across all three waves (see Supplementary Table S1). The scale has a validated Hungarian adaptation, and we have utilized this particular version (Lopez-Fernandez et al., 2018).

#### Mobile phone use while driving

Four items were generated to assess mobile phone use while driving (MPUWD). Participants were asked to rate the frequency of their mobile phone use while driving on a 6-point Likert scale (ranging from 0 = never to 5 = very often). The question of ‘How often do you engage in the following activities while driving?’ had to be answered in relation to four activities: 1. “I use a headset when I'm on the phone”, 2. “Making calls with the phone in hand.”, 3. “Sending messages.”, and 4. “Using social networking sites”. We calculated the total scores for these four items at T1, T2, and T3, and a reliability test indicated that this item set displayed good internal consistency across all waves (see Supplementary Table S1). A higher total score indicates more intensive mobile phone use while driving.

#### Rumination

We utilized the short version of the Ruminative Response Scale (RRS; Kokonyei et al., 2016; Treynor et al., 2003) to evaluate rumination at T1. This scale consists of 10 items and assesses two facets of rumination: brooding (comprising five items, e.g., “Think ‘Why do I always react this way?’”) and reflective pondering (comprising five items, e.g., “Go someplace alone to think about your feelings.”). Respondents were instructed to rate the frequency of these behaviors on a four-point Likert scale, ranging from ‘almost never’ to ‘almost always,’ in response to the question “How often do you…” In this study, we applied only the total score of RRS that showed good reliability (see Supplementary Table S1). A higher score on RRS indicates more intensive ruminative response tendency. The scale has a validated Hungarian adaptation, and we have utilized this particular version (Kökönyei et al., 2016).

#### Anxiety

To measure anxiety symptoms at time T1, we utilized the Anxiety subscale of the Brief Symptom Inventory 18 (BSI-18; Asner-Self, Schreiber, & Marotta, 2006; Franke et al., 2017; Unoka et al., 2004). The BSI-18 is a self-report scale that prompts respondents to evaluate their distress levels over the preceding seven days using a five-point Likert scale, ranging from 0 (not at all) to 4 (extremely). The Anxiety subscale contains 6 items, e.g., “Suddenly scared for no reason.” A higher score indicates a higher degree of anxiety. In the current study, the scale showed good internal consistency (see Supplementary Table S1). The scale has a validated Hungarian adaptation, and we have utilized this particular version (Unoka et al., 2014).

#### Worry

We employed the ultra-brief version of the Penn State Worry Questionnaire (PSWQ) as a measure of trait worry at T2 (Berle et al., 2011; Pajkossy et al., 2015). This abbreviated version, derived from the original 16-item instrument (Meyer et al., 1990), consists of three items (e.g., “Many situations make me worry.”). Participants were asked to rate these items on a Likert scale ranging from 1 (“not at all typical of me”) to 5 (“very typical of me”). Notably, the ultra-brief PSWQ is efficient to administer and score since it lacks reverse-coded items, while still demonstrating similar psychometric properties to the full-length version. A higher score on the PSWQ indicates a higher frequency of worrying. The scale has a validated Hungarian adaptation, and we have utilized this particular version (Pajkossy et al., 2015). Our study confirmed the good internal consistency of this concise version across all three waves (see Supplementary Table S1).

Table 1 provides a summary of the scales used during each data collection period.

**Table 1.** Scales used at each data collection time

	Time 1	Time 2	Time 3
BWAS	✓	✓	✓
PMPUQ-SV;	✓	✓	✓
MPUWD	✓	✓	✓
RRS	✓		
BSI Anxiety	✓		
PSWQ		✓	

*Note*. BWAS, Bergen Work Addiction Scale; PMPUQ-D, Dependency scale of the Problematic Mobile Phone Use Questionnaire; MPUWD, mobile phone use while driving; RRS, Ruminative Response Scale; BSI, Brief Symptom Inventory; PSWQ, Penn-State Worry Questionnaire.

### Statistical analysis

Statistical analyses were performed using IBM SPSS Statistics version 29.0 software, in conjunction with the Hayes' PROCESS Macro extension program. Longitudinal sampling weights were taken into account during the analyses. Multivariate longitudinal weighting of the sample obtained in all three waves was conducted in two phases. Firstly, raw weights were devised through matrix weighting across three variables (gender, expected educational attainment, and religiosity) known to have a significant impact on the binary logistic regression model created to elucidate sample retention, thereby compensating for sample attrition. Subsequently, calibrated item-count matrix weighting was applied based on stratum category to align with the initial sample population distribution across strata (specifically, residence, i.e., districts, and age groups).

After assessing scale reliability and computing basic statistics, we conducted Pearson's correlation analysis to examine the relationships among the variables. Next, we categorized participants into two groups based on their BWAS scale scores in the first wave (T1): those meeting the cut-offs were classified as “at risk of work addiction,” while those scoring below the cut-offs were designated as “not at risk of work addiction”. We compared these two groups using independent samples *t*-tests based on their excessive mobile phone use and mobile phone use while driving at T1, T2, and T3. To explore the role of rumination and anxiety measured at T1 and worry at T2 as mediators between work addiction at T1 and technology use measured at T2, we conducted path analyses, namely a mediation analyses utilizing the PROCESS Macro. In the initial step, the roles of the three mediator variables were tested in individual path models. Subsequently, in the next step, all three mediators were incorporated into a single path model. Multivariate analyses were conducted if the results obtained in the univariate analysis met the specified conditions. For all mediation analyses, the number of bootstrap samples for percentile bootstrap confidence intervals was set at 5,000.

### Ethics

The study protocol was designed in accordance with guidelines of the Declaration of Helsinki and was approved by the Scientific and Research Ethics Committee of the Medical Research Council (approval number 60471-4/2018/EKU).

## Results

Correlation analyses indicate that excessive mobile phone use and mobile phone use while driving were relatively independent factors: mobile phone use while driving, measured at waves 1 and 3, showed no relationship with any of the PMPUQ-D variables, and MPUWD measured at time T2 exhibited a significant, albeit very weak, negative relationship with the PMPUQ-D scale at T1, T2, and T3. We also found that work addiction at T1 has no relationship with excessive mobile phone use at T1. However, it exhibits a statistically significant but notably weak negative relationship with excessive mobile phone use at T2 and T3. In contrast, the symptoms of work addiction measured at time T1 demonstrate a significant positive relationship with excessive mobile phone use while driving across all three datasets. Regarding rumination, anxiety, and worry, all these dimensions showed significant and positive correlation with work addiction in all waves. While rumination and anxiety measured at time T1 exhibited a moderate strength of association, worrying measured at time T2 displayed a weak relationship with T1 work addiction (see Supplementary Table S2).

Independent sample *t*-tests indicate that the at-risk group for work addiction and the non-at-risk group for work addiction did not demonstrate significant differences in terms of excessive mobile phone use at T1 and T2 (Table 2). However, contrary to our assumptions, the at-risk group showed significantly lower scores on the PMPUQ-D scale at T3 when compared to the non-at-risk group, with a medium effect size. However, individuals in the at-risk group reported more frequent mobile phone use while driving at T3 compared to those in the non-risk group. The effect size of the difference was small. No difference was found at time T1 and time 2 (Table 2).

**Table 2.** Differences in excessive mobile phone use and mobile phone use while driving between at-risk and non-at-risk groups of work addiction

	Non-at-risk group of work addiction – T1			At-risk group of work addiction – T1			*t (df)*	*p*	*d*
	*N*	*M*	*SD*	*N*	*M*	*SD*			
T1 PMPUQ-D	1,802	14.61	4.27	59	14.55	3.67	0.093 (1859)	0.926	0.015
T2 PMPUQ-D	1,802	15.36	4.31	59	14.35	3.80	1.777 (1859)	0.076	0.248
T3 PMPUQ-D	1,802	16.39	4.51	59	13.42	4.43	4.966 (1859)	<0.001	0.664
T1 MPUWD	1,802	4.46	4.24	59	4.10	4.56	0.644 (1859)	0.520	0.081
T2 MPUWD	1,802	4.33	3.71	59	5.05	4.84	−1.123 (60.25)	0.266	0.166
T3 MPUWD	1,802	3.96	3.12	59	5.64	4.90	−2.618 (59.55)	<0.001	0.409

*Note*. BWAS, Bergen Work Addiction Scale; PMPUQ-D, Dependency scale of the Problematic Mobile Phone Use Questionnaire; MPUWD, mobile phone use while driving; T1, Time 1; T2, Time 2; T3, Time 3.

Since little or no relationship was observed between T1 work addiction and the PMPUQ-D scale, path model analysis was not conducted with this variable. However, due to the significant positive association between T1 work addiction and T3 MPUWD, all four path models were executed. In the first path model, we examined whether rumination at time T1 plays a mediating role between T1 work addiction and T3 MPUWD. The results indicate that the model fit is good, with the predictor (T1 BWAS) and mediator (T1 RRS) variables collectively explaining 28% (*p* < 0.001) of the total variance in T3 MPUWD. The pathway model reveals a significant partial indirect mediation, where the direct effect accounts for 77.90% of the total effect (*β* = 0.141; *p* < 0.001), and the indirect effect contributes 22.1% (*β* = 0.059; *p* < 0.001) (Supplementary S3 and F1). In the second path model, we explored whether anxiety at T1 mediates the relationship between T1 work addiction and T3 MPUWD. The results indicate that the model fits well, with the predictor (T1 BWAS) and mediator (T1 BSI Anxiety) variables collectively explaining 31.1% (*p* < 0.001) of the total variance in T3 MPUWD. The pathway model reveals significant partial indirect mediation, where the direct effect accounts for 61.88% of the total effect (*β* = 0.113; *p* < 0.001), and the indirect effect contributes 38.12% (*β* = 0.069; *p* < 0.001) (Supplementary S4 and F2). As the third path model shows, worry at time T2 mediates the relationship between T1 work addiction and T3 MPUWD. This model also fits well, with the predictor (T1 BWAS) and mediator (T2 PSWQ) variables collectively explaining 28.4% (*p* < 0.001) of the total variance in T3 MPUWD. The pathway model reveals significant partial indirect mediation, where the direct effect accounts for 92.81% of the total effect (*β* = 0.168; *p* < 0.001), and the indirect effect contributes 7.19% (*β* = 0.013; *p* < 0.001) (Supplementary S5 and Fig. 3).

**Fig. 1.**
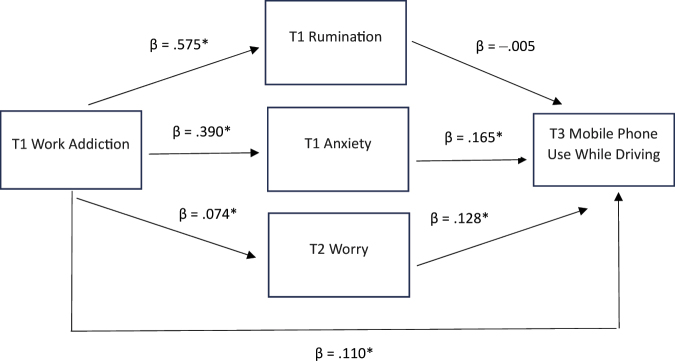
Results of the fourth mediation model incorporating all mediator variables *Note*. *p < 0.001 T1, Time 1; T2, Time 2; T3, Time 3; β, standardized Beta coefficients

Lastly, we conducted a path model analysis by simultaneously incorporating all three mediator variables. In this scenario, only two of the three variables emerged as significant mediators in the relationship between BWAS and MPUWD: T1 anxiety and T2 worry, while T1 rumination did not exhibit significant mediation. The predictor (T1 BWAS) and the mediator variables, when considered together, explain 31.9% (*p* < 0.001) of the total variance in T3 MPUWD. The pathway model demonstrates a significant partial indirect mediation, with the direct effect accounting for 60.78% of the total effect (*β* = 0.118; *p* < 0.001), and the indirect effect contributing 39.22% (*β* = 0.071; *p* < 0.001) (Table 3, Fig. 1). Among the mediators, anxiety exhibited the most substantial effect (*β* = 0.065; *p* < 0.001; 35.91% of the total effect).

**Table 3.** Results of the fourth mediation model incorporating all mediator variables

Path	Beta	*SE*	LLCI	ULCI	*p*
T1 Work Addiction → T1 Rumination	0.575	0.020	0.535	0.614	<0.001
T1 Work Addiction → T1 Anxiety	0.390	0.014	0.362	0.419	<0.001
T1 Work Addiction → T2 Worry	0.074	0.009	0.056	0.093	<0.001
T1 Rumination → T3 Mobile Phone Use While Driving	−0.005	0.021	−0.046	0.036	0.801
T1 Anxiety → T3 Mobile Phone Use While Driving	0.165	0.029	0.108	0.223	<0.001
T2 Worry → T3 Mobile Phone Use While Driving	0.128	0.038	0.054	0.202	<0.001
T1 Work Addiction → T3 Mobile Phone Use While Driving	0.110	0.019	0.074	0.147	<0.001

Effect	Beta	*SE*	LLCI	ULCI	% mediation
Indirect Total	0.071*	0.015	0.042	0.101	39.22%
T1 Rumination	−0.003	0.014	−0.030	0.026	
T1 Anxiety	0.065*	0.015	0.035	0.094	
T2 Worry	0.009*	0.004	0.002	0.019	
Direct	0.118*	0.018	0.083	0.156	60.78%
Total	0.181*	0.015	0.151	0.211	100%

*Note*. *p < 0.001;

T1, Time 1; T2, Time 2; T3, Time 3; SE, standard error; LLCI, lower level of the 95% confidence interval; ULCI, upper level of the 95% confidence interval.

## Discussion

In our longitudinal study, which involved a representative sample of young adults in a European capital city, we investigated the relationship between work addiction and mobile phone usage. Specifically, we investigated whether the presence of work addiction symptoms predicted future heavy mobile phone use, including hazardous behaviors such as texting or using social media while driving. Surprisingly, our findings did not show any association between work addiction and either current or future intensive mobile phone usage. Workaholics, when compared to non-workaholics, did not exhibit a stronger attachment to their mobile phones or a feeling of dependence. Interestingly, individuals without work addiction were more likely to exhibit symptoms of mobile phone dependence over time. However, the situation was different when it came to hazardous mobile phone, i.e., using it while driving. The group at risk for work addiction consistently displayed higher mobile phone usage habits while driving. Although we must exercise caution in establishing causal relationships, our longitudinal design suggests that a stronger presence of work addiction symptoms predicts future hazardous mobile phone use, such as using a mobile phone while driving. This trend was apparent in both our univariate and multivariate analyses.

The personality of individuals with work addiction can significantly influence the interpretation of the results. In the case of work addiction, individuals often find it imperative to remain constantly available and receive information promptly. They typically grapple with a shortage of time to accomplish tasks, often feeling perpetually rushed (Sussman, 2012). This tendency is frequently linked to an inclination to be overly controlling (Mudrack & Naughton, 2001; Van Beek et al., 2011), making it challenging for them to delegate tasks to others (Robinson et al., 1992; Scott et al., 1997). They are characterized by obsessive traits (Butucescu & Uscătescu, 2013; Mudrack, 2004); therefore, they exhibit heightened responsibility and a relentless pursuit of perfection. They meticulously review their work, often face challenges in making decisions, yet once they do decide, they remain resolutely committed. It is also because of their high perfectionism (Clark et al., 2010; Stoeber et al., 2013) and conscientiousness (Andreassen, Hetland, & Pallesen, 2010) that they have to take every opportunity to get on with their work. Driving time is an excellent opportunity to do this, and they can feel that the time is useful, rather than wasted sitting around doing ‘nothing’. This behavior may resemble the ‘fear of missing out’ phenomenon associated with social media use (Fioravanti et al., 2021), where individuals fear missing out on social experiences. In the context of using technological tools, there may also be a fear of missing important information, messages, or notifications. Our results also indicate that social media use occurs while driving, further supporting this assumption. However, future research should explore the relationship between work addiction and problematic social media use, which remains an area yet to be thoroughly understood.

Workaholics also struggle to maintain a clear boundary between work and personal life, often finding that the demands of work spill into their personal time (Aziz et al., 2010). While there are occasions when driving during working hours or for work-related reasons is necessary, in most cases, it occurs outside of regular working hours. Workaholics, however, have a harder time disengaging from their work, and thus, using the mobile phone while driving becomes an opportunity to extend their work hours. It is essential to consider that work addiction is significantly influenced by organizational factors too, such as workplace culture and organizational expectations. The challenge that a worker encounters when trying to detach from work and their inclination to stay connected on the mobile phone even while driving may be attributed to the workplace's demanding expectations for constant availability and rapid task completion, i.e., they impose exceptionally high work demands on the employee (Molino et al., 2016). Excessively high expectations, extreme workloads, and destructive competition all contribute to workaholic behavior (Guglielmi et al., 2012; Porter, 1996). Additionally, an excessive organizational identity also poses a risk factor for workaholism (Avanzi, van Dick, Fraccaroli, & Sarchielli, 2012). These factors, originating within the organization, might also intensify the compulsion to use the mobile phone even while driving.

Our research has also revealed that the relationship between work addiction and heightened mobile phone use while driving is mediated by rumination, anxiety, and increased worrying. In line with previous studies (Kun, Takacs, et al., 2020a, b), our findings affirm that workaholics tend to exhibit more negative emotional states, including anxiety, rumination, and worrying, which can spill over into other maladaptive behaviors like using mobile phones while driving. From our analysis, we conclude that the presence of work addiction escalates subsequent mobile phone use while driving because individuals find it challenging to detach from their mobile phones even while driving due to these negative emotions and perseverative cognitions. While we can only provide a general overview of the nature of these fears, concerns, and worries, it is plausible that their content primarily revolves around work-related issues, tasks, or work-life conflicts (Maslach, 1986; Williamson & Clark, 2017). Further research is necessary to explore this aspect in greater detail, such as investigating the relationship between work-related rumination (Cropley & Zijlstra, 2011) and work addiction, a topic that remains relatively unexplored.

The results of our fourth path analysis also indicated that negative emotions concerning the future, namely anxiety and worry, played a more prominent mediating role in the relationship between work addiction and driving mobile phone use compared to ruminations about the past. The latter ceased to be a significant mediator when all three factors were included simultaneously in the model. In other words, mobile phone use while driving may be primarily linked to an individual's inability to distance themselves from their mobile phone. This could be attributed to perfectionist traits or high conscientiousness (Falco et al., 2020; Mazzetti et al., 2014), as they may fear falling short of lofty expectations. Consequently, it appears to be less connected to reflecting on past events and repeatedly reaching for the mobile phone. Nevertheless, it is important to note that the elimination of rumination's role as a mediator could also stem from its strong positive correlation with anxiety.

While our research was conducted using a representative sample of young individuals in a capital city and employed a longitudinal design, several limitations should be acknowledged. Firstly, our research relied on self-report questionnaires, which can introduce biases related to social desirability, self-knowledge, and memory recall. Secondly, we lack formal diagnostic criteria and a corresponding measurement tool for work addiction, thus relying on a reliable screening test to identify individuals at high and low risk. Thirdly, we employed custom items to assess mobile phone use while driving, and although we conducted internal reliability testing that yielded satisfactory results, it would be advisable in future research to utilize a psychometrically validated scale for this specific purpose.

In conclusion, our current study indicates that work addiction is a predictor of problematic (dangerous) mobile phone use, as opposed to merely intensified use. This problematic mobile phone use is further associated with heightened levels of anxiety and worry. Our research contributes to the growing body of evidence highlighting the maladaptive aspects of work addiction and underscores the need for enhanced prevention and treatment strategies for this phenomenon.

## Funding sources

This study was supported by the Hungarian National Research, Development and Innovation Office (Grant numbers: KKP126835, FK134807, K143764). GK was supported by the Hungarian Brain Research Program 3.0 (NAP2022-I-4/2022).

## Authors' contribution

Conceptualization: BK, BP, ZD; methodology: BK, AE, BP, ZD; formal analysis and investigation: BK, BP; writing - original draft preparation: BK; writing – review and editing: BK, BP, AE, GK, ZD; funding acquisition: ZD; resources: BK, ZD; supervision: BP, GK, ZD.

## Conflict of interest

ZD is the Editor-in-Chief of the Journal of Behavioral Addictions, and GK is an Associate Editor in the Journal of Behavioral Addictions. All the other authors declare no conflict of interest.

## Supplementary material
