# Supplementary material for: Driving and mobile phone use: Work addiction predicts hazardous but not excessive mobile phone use in a longitudinal study of young adults
Source: J Behav Addict. 2024 Mar 8;13(1):66–75. doi: 10.1556/2006.2024.00007 (PMC10988412; doi:10.1556/2006.2024.00007)
Supplement: Supplementary file 1 [file jba-13-066-s001.docx]

**Kun, B. et al.: Driving and mobile phone use: Work addiction predicts hazardous but not excessive mobile phone use in a longitudinal study of young adults**

[**https://doi.org/10.1556/2006.2024.00007**](https://doi.org/10.1556/2006.2024.00007)

**Supplementary Table S1.**

*Basic Statistics and Reliability Scores of the Variables*

|  | Range | *M (SD)* | Cronbach’s alpha |
| --- | --- | --- | --- |
| T1 BWAS | 0-28 | 4.19 (4.81) | .860 |
| T2 BWAS | 0-28 | 4.26 (4.90) | .817 |
| T3 BWAS | 0-28 | 5.23 (6.46) | .943 |
| T1 PMPUQ-D | 5-20 | 14.46 (4.17) | .878 |
| T2 PMPUQ-D | 5-20 | 16.02 (4.46) | .887 |
| T3 PMPUQ-D | 5-20 | 15.15 (4.21) | .913 |
| T1 MPUWD | 0-20 | 4.53 (4.40) | .882 |
| T2 MPUWD | 0-20 | 4.32 (3.88) | .878 |
| T3 MPUWD | 0-20 | 3.93 (3.21) | .900 |
| T1 RRS | 10-40 | 14.37 (5.15) | .914 |
| T1 BSI-18 Anxiety | 6-30 | 8.18 (3.68) | .914 |
| T2 PSWQ | 3-15 | 4.16 (1.95) | .879 |

*Note*. BWAS, Bergen Work Addiction Scale; PMPUQ-D, Dependency scale of the Problematic Mobile Phone Use Questionnaire; MPUWD, mobile phone use while driving; RRS, Ruminative Response Scale; BSI, Brief Symptom Inventory; PSWQ, Penn-State Worry Questionnaire; T1, Time 1; T2, Time 2; T3, Time 3.

**Supplementary Table S2**

*Pearson’s Correlation Coefficients Between Study Variables*

|  | 1 | 2 | 3 | 4 | 5 | 6 | 7 | 8 | 9 | 10 | 11 |
| --- | --- | --- | --- | --- | --- | --- | --- | --- | --- | --- | --- |
| 1. T1 BWAS | – |  |  |  |  |  |  |  |  |  |  |
| 2. T1 BWAS | .398** | – |  |  |  |  |  |  |  |  |  |
| 3. T3 BWAS | .268** | .338** | – |  |  |  |  |  |  |  |  |
| 4. T1 PMPUQ-D | –.043 | .005 | .130** | – |  |  |  |  |  |  |  |
| 5. T2 PMPUQ-D | –.067** | –.069** | .144** | .387** | – |  |  |  |  |  |  |
| 6. T3 PMPUQ-D | –.134** | –.056* | .129** | .432** | .577** | – |  |  |  |  |  |
| 7. T1 MPUWD | .061* | .002 | –.037 | –.012 | –.026 | –.030 | – |  |  |  |  |
| 8. T2 MPUWD | .188** | .180** | .076** | –.112** | –.063** | –.076** | .152** | – |  |  |  |
| 9. T3 MPUWD | .260** | .132** | .159** | –.049* | .051* | .003 | .113** | .473** | – |  |  |
| 10. T1 Rumination | .579** | .402** | .262** | –.039 | –.044* | –.131** | .057* | .114** | .220** | – |  |
| 11. T1 Anxiety | .554** | .375** | .264** | –.008 | –.015 | –.090** | .073** | .153** | .263** | .726** | – |
| 12. T2 Worry | .194** | .361** | .156** | –.101** | –.058* | –.041 | .034 | .187** | .138** | .218** | .242** |

*Note*. BWAS, Bergen Work Addiction Scale; PMPUQ-D, Dependency scale of the Problematic Mobile Phone Use Questionnaire;

MPUWD, mobile phone use while driving; T1, Time 1; T2, Time 2; T3, Time 3.

* *p* < .05; ** *p* < .01

**Supplementary Table S3**

*Results of the First Path Model: the T1 Rumination Mediates the Relationship Between T1 Work Addiction and T3 Mobile Phone Use While Driving*

| Path | Beta | *SE* | LLCI | ULCI | *p* |
| --- | --- | --- | --- | --- | --- |
| T1 Work Addiction 🡪 T1 Rumination | .575 | 0.020 | 0.535 | 0.614 | <.001 |
| T1 Rumination 🡪 T3 Mobile Phone Use While Driving | .070 | 0.017 | 0.035 | 0.104 | <.001 |
| T1 Work Addiction 🡪 T3 Mobile Phone Use While Driving | .181 | 0.015 | 0.151 | 0.211 | <.001 |
| Effect | Beta | *SE* | LLCI | ULCI | % mediation |
| Indirect | .059* | 0.018 | 0.023 | 0.095 | 22.10% |
| Direct | .141* | 0.018 | 0.105 | 0.177 | 77.90% |
| Total | .181* | 0.015 | 0.151 | 0.211 | 100% |

*Note*. * *p* < .001

T1, Time 1; T2, Time 2; T3, Time 3; SE, standard error; LLCI, lower level of the 95% confidence interval; ULCI, upper level of the 95% confidence interval.

**Supplementary Table S4**

*Results of the Second Path Model: the T1 Anxiety Mediates the Relationship Between T1 Work Addiction and T3 Mobile Phone Use While Driving*

| Path | Beta | *SE* | LLCI | ULCI | *p* |
| --- | --- | --- | --- | --- | --- |
| T1 Work Addiction 🡪 T1 Anxiety | .390 | 0.014 | 0.363 | 0.419 | <.001 |
| T1 Anxiety 🡪 T3 Mobile Phone Use While Driving | .176 | 0.024 | 0.129 | 0.223 | <.001 |
| T1 Work Addiction 🡪 T3 Mobile Phone Use While Driving | .181 | 0.015 | 0.151 | 0.211 | <.001 |
| Effect | Beta | *SE* | LLCI | ULCI | % mediation |
| Indirect | .069* | 0.014 | 0.042 | 0.095 | 38.12% |
| Direct | .113* | 0.018 | 0.078 | 0.147 | 61.88% |
| Total | .181* | 0.015 | 0.151 | 0.211 | 100% |

*Note*. * *p* < .001

T1, Time 1; T2, Time 2; T3, Time 3; SE, standard error; LLCI, lower level of the 95% confidence interval; ULCI, upper level of the 95% confidence interval.

**Supplementary Table S5.**

*Results of the Third Path Model: the T2 Worry Mediates the Relationship Between T1 Work Addiction and T3 Mobile Phone Use While Driving*

| Path | Beta | *SE* | LLCI | ULCI | *p* |
| --- | --- | --- | --- | --- | --- |
| T1 Work Addiction 🡪 T2 Worry | .074 | 0.009 | 0.056 | 0.093 | <.001 |
| T2 Worry 🡪 T3 Mobile Phone Use While Driving | .172 | 0.038 | 0.098 | 0.246 | <.001 |
| T1 Work Addiction 🡪 T3 Mobile Phone Use While Driving | .181 | 0.015 | 0.151 | 0.211 | <.001 |
| Effect | Beta | *SE* | LLCI | ULCI | % mediation |
| Indirect | .013* | 0.005 | 0.005 | 0.023 | 7.19% |
| Direct | .168* | 0.015 | 0.138 | 0.199 | 92.81% |
| Total | .181* | 0.015 | 0.151 | 0.211 | 100% |

*Note*. * *p* < .001

T1, Time 1; T2, Time 2; T3, Time 3; SE, standard error; LLCI, lower level of the 95% confidence interval; ULCI, upper level of the 95% confidence interval.

**Supplementary Figure F1**

*Results of the First Path Model Testing the Mediator Effect of T1 Rumination between T1 Work Addiction T1 and T3 Mobile Phone Use While Driving*

β = 0.575*

T1 Rumination

T3 Mobile Phone Use While Driving

β = 0.070*

T1 Work Addiction

β = 0.181*

*Note*. * *p* < .001

T1, Time 1; T2, Time 2; T3, Time 3; β, standardized Beta coefficients

**Supplementary Figure F2**

*Results of the Second Path Model Testing the Mediator Effect of T1 Anxiety Between T1 Work Addiction T1 and T3 Mobile Phone Use While Driving*

β = 0.390*

T1 Anxiety

T3 Mobile Phone Use While Driving

β = 0.176*

T1 Work Addiction

β = 0.181*

*Note*. * *p* < .001

T1, Time 1; T2, Time 2; T3, Time 3; β, standardized Beta coefficients

**Supplementary Figure F3**

*Results of the Third Path Model Testing the Mediator Effect of T2 Worry Between T1 Work Addiction and T3 Mobile Phone Use While Driving*

β = 0.074*

T2 Worry

T3 Mobile Phone Use While Driving

β = 0.172*

T1 Work Addiction

β = 0.181*

*Note*. * *p* < .001

T1, Time 1; T2, Time 2; T3, Time 3; β, standardized Beta coefficients
